# A Reply to the Attacks of Dr. Charles Caldwell

**Published:** 1850-06

**Authors:** Lunsford P. Yandell

**Affiliations:** Louisville, Ky.


### A REPLY

TO THE

### ATTACKS OF DR. CHARLES CALDWELL.

BY

LUNSFORD P. YANDELL.

LOUISVILLE, KY.
PRENTICE AND WEISSINGER.
1850.

### DR. YANDELL'S REPLY TO DR. CALDWELL.

I find myself placed in strange circumstances. An old friend, with whom I have cooperated in many enterprises, with whom I have lived long in the interchange of kind offices, whom I have often praised and defended, has become my defamer. I am about to reply to an envenomed personal attack lately made upon me in a public print by Dr. Charles Caldwell. I confess, on account of the age of my assailant, the character of his attack, and the relations which have subsisted between us, I do so with extreme repugnance. I am not without misgivings as to the propriety of making any reply. The charges are so vague and contradictory, the spirit and temper of the attack are so unbecoming, the style in which it is made is so feeble, the production, altogether, is so pitiably imbecile, that my friends here generally think that I might safely leave it to its own sure fate. But in consideration of the position which my assailant once held in his profession and of our long association in office, I have concluded that I ought not to let such charges pass unnoticed, however driveling the manner in which they are made.

The occasion of this assault upon me by Dr. Caldwell is his removal from the chair which he formerly held in the University of Louisville. He professes to believe that it was through my influence with the Board of Trustees that he was removed. The board saw fit to transfer me to the chair which he had occupied, and he assumes that, therefore, the members were my dupes and tools. He charges that I neglected the duties of my chair, as, in letters to the faculty and board, last spring, he asserted, that I had "disgraced it for twenty years," and yet he contends that by my intrigues with the board I was able to accomplish his overthrow. And all this I did, as he expresses it, after he had "rescued me from obscurity, and elevated me to the chair I then held, and by his patronage and uninterrupted favors, extending through nearly an average lifetime, had contributed to sustain me in it."

These are his charges. They are vaguely stated in his "Story"—rather insinuated than asserted; but some of them have been long in circulation; they have been repeated for months past wherever Dr. Caldwell has gone, and have been propagated industriously by his friends in low, abusive handbills.

It is not a new story with Dr. Caldwell, that he "rescued me from poverty and obscurity." This boast he made a number of years ago, and under circumstances which will throw some light upon his present attitude towards me. In the spring of 1843, he published an unlucky "critique" upon Liebig's Animal Chemistry, which, as editor of a medical journal, I felt it to be my duty to notice. My review, though less severe than the demerits of the work deserved and much lest severe than the strictures of

About that time he had bought a printing press f creature of his, one *William Newton*—well rem bered in Lexington and not altogether forgotte Louisville—and had set his pet up as editor in Ev ville. The first number of the paper of this para and *special* favorite of Dr. Caldwell contained the lowing paragraph:

"Professor Caldwell had been to him (Profe Yandell) a friend and a father. He had instru him in his ignorance—supported him in his w ness—supplied him in his poverty—and defen him in his need. But, notwithstanding all this, v do we see? With a shameless ingratitude that receive no excuse, he turned his pen against benefactor," &c.

From all this, the reader would conclude th had been a *beneficiary*—a regular *charity-pupi* Dr. Caldwell's; and this is just the impressio has sought to make whenever he has spoken of for a good while past.

Poverty is no crime. If it had been my lot t born poor I trust I should have been above the w ness of wishing to conceal it. But I was not b so; for my venerated father, though not a man great wealth, was a physician of great popular and commanded in his profession, for many ye and to the end of his life, a large and profitable siness. He was, moreover, as a great physician n be, of an enlarged and liberal spirit, and strove give his children a thorough education; so that d ing my minority I enjoyed every advantage that ney could bestow. He sent me to Lexington in fall of 1823, and I attended Dr. Caldwell's lectu but I also paid him for them. Besides his pul ticket of $20, he had a private ticket, for whi also paid him $20. In the course of that winter published the first edition of his "Elements of Pl nology," and, by reference to a diary which I k at the time, I see that I subscribed and paid him *ten copies* of that work. So much for the charge having been his "beneficiary"—a charge so conte tible that I should have deemed it unworthy of tice if it had not been coupled with others of a m serious character. Dr. Caldwell himself would h scrupled to make it, if he had not felt that it was cessary to give point and keenness to his other cusations.

But Dr. Caldwell claims to have "rescued" "from obscurity," and "elevated" me "to the chair lately held, and "by his patronage and uninterrup favors, extending through nearly an average li time, contributed to sustain me in it." In anotl place he says, I "neglected to labor" in this chair which he "elevated" me, and in which he so lo sustained me. In the streets, on the highway as traveled, and in official letters to the faculty and the boardof trustees, he has asserted in plain ter

*rs.*" I might stop here for a moment and com- 'e these two statements together. It might be a astion how far a professor acted in good faith to titutions which he had sworn to support, when he pt an unworthy colleague in office so long. But I ve this to be settled by Dr. Caldwell, and proceed notice his boast of "rescuing" me "from obscu- y."

t is true that when I set out in my profession I s obscure, precisely in the sense in which every ung professional man is obscure. But it was not g, as Dr. Caldwell knows, before I had succeed- to the large and lucrative business of my father, d was therefore at least independent. My repu- ion, humble as it was, in a few years extended be- nd the county in which I lived, and, as Dr. Cald- ll also knows, I had been on several occasions led to neighboring counties to perform difficult erations in surgery long before I was invited to school in Lexington. I had become a contribu- to some of the leading papers in my native State, d a correspondent of more than one medical jour- . I do not pretend that there was in those early asions much to be praised, but I happen to have fore me an expression of the opinion they led *Dr. ldwell* to form of my abilities as a writer. In a ter dated December 18th, 1827, he thus speaks of o of my earliest efforts: "Your very excellent and gant papers have been received, and submitted mediately to the inspection of our editors. I need rcely say to you that they concur with me most rfectly in my estimate of the articles. Indeed not s to concur would prove them to be wanting alike scientific judgment and literary taste. For with the slightest disposition to flatter, or to express idea which I do not honestly believe, permit me assure you, that, if I have any competency to lge, the papers are both written with an amount intellect and in a style and manner that would be norable to any pen in our country, and creditable d advantageous to *any* journal in *any* country."

n reference to the same papers, which I had re ested him to correct if he discovered any inaccura- s in them, he remarked, in a letter dated a few days bsequently: "Your two papers in our possession ed no correction. *Alter* them I *could; amend* them I *ld not. Possibly* in correcting the press, I may nge a *word* or a *point*; but *probably* I shall deem n that unnecessary. I shall not *supererogate.*" e italics, in both extracts, are Dr. Caldwell's.

n the same letter he says:

'I forwarded to my friend, President Cooper, of uth Carolina, a copy of my Elements. I think it bable that he will make a paper out of it for the uthern Review. It is right you should know this, I confess myself ambitious that the *best* review t should appear in our own journal. Cooper is a werful writer; but your superior knowledge of the ence gives you the advantage. I feel persuaded, refore, that you *can* produce the best paper. But u must *labor* for it. *Common* writing and analysis l not do to compare with those of Cooper. He is gether *uncommon,* both in intellect and *scholar- p.*" The italics again are Dr. Caldwell's.

Of an address which I delivered in Nashville, in the fall of 1830, he thus spoke, in a letter dated Dec. 5th:

"Your address I consider the best production I have seen from your pen. Indeed I know not how it could be improved. Added to as good a selection of topics and points, as I think can be made, its spirit is animated, its tenor lofty, its strain eloquent and impressive, and its diction correct. And what mor, would you have? It is rich in appropriate matter and in manner neither tame nor mad—*ibis tutissimus medio*—and that is what it ought to be."

In the spring of 1830 the Medical Society of Tennessee, composed of delegates elected by the Legislature from all parts of the State, held its first meeting in Nashville. Dr. Caldwell was present at that meeting. That distinguished body of physicians did me the honor to elect me Corresponding Secreta- ry of the Society. I record the fact here, because, in the circumstances attending it, I remember it as one of the most grateful honors ever conferred upon me by a profession to which I am so largely indebted.

The autumn ensuing, having removed to Nash- ville, I was conferring with my friends (among the rest, the Hon. John Bell, President Lindsley, and Dr. Overton) on the expediency of attempting a medical school in that city, and wrote to Dr. Cald- well asking his judgment in the matter. His reply was prompt and explicit. The scheme, which I had deeply at heart and which was favored by several of my friends, met with his decided reprobation. He says, thus emphatically:

"In the abstract, the scheme is unfriendly, *in fact*, to medical teaching, and therefore to medical science in the West. We are not yet ripe for *three* schools. Our population is not sufficient to maintain them in such numbers and standing as to make them objects of *attention* and *ambition* to competent men. They will necessarily dwindle in *strength* and *merit.* In stead of Atlantic pupils coming to *them*, as now, all western pupils of generous ambition, and who can command the means, will repair to the eastern schools. Western teaching will become a mockery. I repeat, then, that *should* a medical school be erect- ed in Nashville, either *it* must fail, or *western medical teaching* will fail. I say definitely, then, that the measure is unfriendly, *in fact,* to the profession in the West. Can it not be postponed, at least, until society is ripe for it, or arrested for the present alto- gether?"

But the most significant part of his letter is behind, and reads thus:

"Our chemical chair has fallen into such a condi- tion as to call imperatively for reformation and ad- ditional strength; and you are looked to, at present, for that purpose. * * * Tell me frankly, then, and as speedily as you can, could you not, without robbing yourself of too much of your professional time, prepare yourself, in a year from this date, to deliver lectures on chemistry, creditable to yourself and useful to others?

"Do you tell me that chemistry is not the branch of science for which you have the greatest predilection? I reply that I know it. Chemistry was never *my* first choice, any more than it is yours; yet when I was perhaps under your age, I delivered in Philadelphia a number of chemical lectures which were pronounced among the best, if not *themselves* the best, that had been listened to in the place. In the course of my experiments I inflamed charcoal with nitric acid, the *first time* it had ever been done in the United States, and after Woodhouse had repeatedly failed to do it, and had even pronounced it *impracticable.* Can you not, then, I repeat, prepare yourself on this branch," &c?

In a letter on the same subject, dated February 11, 1831, he thus wrote—

"The chair must be strengthened. The necessity for it acquires *every hour* the growth and strength of a *whole week.* And you are the only man thought of, or even dreamt of, by the faculty."

Dr. Caldwell's wishes were gratified, and I was placed in the chair. The following notice of my appointment appeared from his pen, shortly afterwards in a Nashville paper:

"Another measure has been recently adopted by that institution (Transylvania University) which, it is believed, will be important. The professor of chemistry, whose knowledge of medicine was but limited, has retired, and Dr. Yandell, of Nashville, Tennessee, a young physician of fine talents and high promise, has been appointed to succeed him. That he is *well and favorably known in the West,* appears from his election to so important a chair. But *he is also known in the East* from his writings, which show him to be a scholar, as well as a man of genius. It cannot be doubted that the change will be beneficial to the school."

This was the obscurity, Dr. Caldwell himself being judge, in which his letter found me—"the *joint* letter," as he expressed it, of himself and three of his colleagues—inviting me to Lexington to "*strengthen the chemical chair*" in Transylvania University. The reader will form his own conclusions as to the motives which prompted to this invitaton. These prudent, wise, discreet professors may have felt a benevolent desire to "rescue" a young man from "obscurity;" but I submit whether it is not quite as probable that they had it in their minds, while they "strengthened" their chemical chair, to "arrest" the enterprise at Nashville and conciliate a State which, next to Kentucky, was furnishing the largest number of students to their school?

But after "elevating" me to the chair, Dr. Caldwell charges that I "disgraced" it. In noticing this charge, I must again permit Dr. Caldwell to answer himself.

He did me the honor to attend my first course of lectures in Lexington, and as he listened to them with much apparent attention and interest he ought to have known something about their merits. Early in the session, writing to a common friend, the late Dr. Becton, of Tennessee, he remarked of me: "Dr. Yandell is doing his duty in fine style and growing rapidly popular." Dr. Caldwell's students of that period will long remember how his views were all at once changed respecting the value of chemical knowledge, and how fervid were his eulogies on the young professor of chemistry. Some of them reside in this city and have favored me with their reminiscences of the winter of 1831–'2. Dr. Bell retains a lively recollection of the incidents of that winter. Dr. Caldwell, as all his earlier pupils know, had been in the habit of speaking lightly of chemistry. "It is an *uncertain* science," he was in the habit of saying—"*far less reliable than phrenology.*" He had penned in his outlines, the textbook of his class, some allusions to it which showed how it was held in his estimation. The students, says Dr. Bell, were curious to see what the Professor would make of these passages now. There they stood in his book before him. When he came to them, instead of the jests with which he had been wont to set the class in a roar, he broke forth into a warm panegyric on the course of lectures which was elevating chemistry in that institution to the dignity of a science. In one word, it was plain that Dr. Caldwell esteemed this, my first course of lectures, *nearly* equal to that course of his in Philadelphia, in which he performed the famous feat of inflaming charcoal by nitric acid. Nor was this all. So effectual was his applause of my lectures, that Dr. Richardson, who had opposed my election, was brought over by it, and was obliged to confess that I had "*reinstated* chemistry." And in the spring following, if Dr. Bell remembers right, these gentlemen could not sufficiently express their admiration at the extent and accuracy of the chemical knowledge displayed by the candidates in their examinations before the faculty.

Such, at least in the estimation of Dr. Caldwell, was the character of my first course of lectures in Lexington. Under the new organization, the number of students increased, and the institution continued to prosper up to the period of the dissolution of the faculty in 1837.

Dr. Dudley, for a long time, had been complaining to his colleagues of the difficulty of procuring subjects for his demonstrations in anatomy, and in the fall of 1835 he declared that the evil had become insupportable. He was obliged, he said, to dwell for weeks on the bones because he was without a subject for demonstrating the soft parts. Nay, he was obliged to persuade students not to dissect, in order to prevent the discovery that he had no subjects for dissection. If the school remained in Lexington, to his mind it was clear that it was "destined to *fritter.*" The effect of such a confession upon the minds of his colleagues can be easily conceived. It was plain to them that if they would sustain themselves they must get to a point where subjects could be obtained, and with one consent they formed the resolution to endeavor to effect the transfer of the medical department of Transylvania University to Louisville. When the scheme came to be publicly discussed, the citizens of Lexington were found to be violently opposed to it. Dr. Dudley deemed it politic to avoid the storm he had raised, and so protested that he had always been opposed to the plan of removal. Dr. Caldwell was a most available scapegoat on such an occasion. He had never been a favorite in Lexington, and, as it could be shown that he had been active in promoting the transfer, it was easy to turn the tide of popular indignation against him. This, for a time, was done most effectually, but to some of us it appeared ungenerous and unjust that the oldest member of the faculty, and not the most popular, should bear the whole odium of a measure in which all his colleagues had participated. We declared, therefore, to the medical class and in all private circles, that all the professors had advocated the transfer of the school, but that the project was first proposed by Dr. Dudley and was especially his. The issue was, that Dr. Dudley quarreled with several of his colleagues, and preferred charges against Dr. Caldwell and myself before the Board of Trustees. The Board dismissed Dr. Caldwell, and, in the hope of restoring harmony among the remaining members, dissolved the faculty.

Preparatory to reorganizing, several leading members of the Board urged me to agree to accept the chair of the Institutes of Medicine, from which Dr. Caldwell had been displaced. My friends in Lexingington, in Tennessee, and the few I then had in Louisville generally concurred in advising me not to connect myself with the enterprise projected in this city. Their belief was that the scheme was nearly hopeless, and that I would suffer pecuniary loss by embarking in it. But my resolution was taken. I could not think of remaining in Lexington after having heard Dr. Dudley's fatal confessions as to the state of his department, and I felt bound to lend Dr. Caldwell whatever aid I could after he had been, as I conceived, so badly treated by the Trustees in Lexington. Dr. Caldwell came to Louisville and made a speech to the citizens. He told them that he had been young but now was old, and that he had, at last, found out that *honesty is the best policy*. He had been many years, in violation of the dictates of conscience, laboring to sustain the school at Lexington, but had now come to labor, in good faith, in behalf of one in Louisville. To the Board of Managers of the Louisville Medical Institute he addressed substantially the same words. The citizens and Board of Managers took up the work with spirit and resolved to establish a school of medicine on the most liberal footing. Dr. Caldwell says he was "*premier*." All the appointments, according to him, were made at his recommendation,

Now, if I had been disgracing the chair I held in Lexington, as Dr. C. asserts I did, was not this a favorable time for correcting the abuse? But did he seek to do it? On the contrary, my friends, Drs. Short and Cooke, can testify how deep and intense was his anxiety that we should all unite with him in the new enterprise. His letters, written to me at the time, are full of expressions showing how important he regarded this to the success of the school. "To destroy the school at Lexington beyond revival," he said, "nothing else is necessary than that Cooke, Short, and yourself be transferred to the school of Louisville." On the 22d of April, 1837, he announced to me, and, as he said, with *great pleasure*, my *unanimous* election to the chair of chemistry in the Louisville Medical Institute. He added: "Your presence in Louisville throughout the summer will be *important* if not *essential*. The field must be occupied by men of *action*—men to ride the whirlwind and breast the troubled wave. If you were here, I would set out for the East in two or three days."

I accepted the chair thus tendered to me. I should have accepted it, under the circumstances, if I had considered the prospects of the school far worse than they seemed to me. They seemed to me all that could be desired. Dr. Caldwell heard my first lecture in Louisville, and he wrote respecting it: "To pronounce it a brilliant effort would be a meagre compliment. It was the vigorous outpouring of a gifted, rich, and cultivated mind. It manifested as much of sound judgment and professional attainment as of imagination, scholarship, and taste; and it abounded in them all. Its most valuable element, however, was the *spirit of truth* with which it was instinct from beginning to end." The Italics everywhere in these quotations are Dr. Caldwell's.

Dr. Short having decided to remain for the time in Lexington, at the suggestion of Dr. Caldwell and Dr. Miller, which met my approbation, I was transferred by the Board of Managers to the chair of materia medica, and the following winter, the chair of chemistry being vacant, I discharged the duties of both professorships. In the spring it was understood that the valuable services of Dr. Short could then be obtained, and I cheerfully consented to return to the chair of chemistry in order that we might introduce into the school so estimable a colleague. A unanimous vote was passed by the faculty recommending the transfer to the board, and thus, by Dr. Caldwell's advice, I was placed a *third time* in the chemical chair.

Finally, on this point. In the autumn of 1846, Dr. Caldwell delivered the public introductory lecture for the faculty to the class. The Louisville Medical Institute had been merged in the University of Louisville. As a separate, independent institution it had ceased to exist. Dr. Caldwell undertook to pronounce its funeral oration, and it was a funeral oration such as would have come from no pen but Dr. Caldwell's. Such a school as the late Medical Institute of Louisville, he declared, the world had never seen. He exhausted his epithets of praise upon it, and, when his eulogy was ended, he declared to some of our common friends that "he could hardly resist the impulse to turn round and take Yandell by the hand and say, *Sir! you and I did all this. You and I made the school.*"

After this fashion it has been, if Dr. Caldwell may be credited, that I disgraced the chair of chemistry so long. But I hasten to conclude this vindication by noticing Dr. Caldwell's last and most serious accusation—that of having by my intrigues with the Board of Trustees brought about his removal from the school. He assigns no motive for this conduct in his published account of the matter, but he has been in the habit of saying that I procured his dismissal in order that I might succeed to his chair.

The charge is [illegible]

honorable gentlemen composing the Board of Trustees. It is an insult to common sense. Who constituted the board against which Dr. Caldwell brings this accusation? James Guthrie, W. S Vernon, James Marshall, S. S. Nicholas, W. F. Bullock, G. W. Weissinger, Isaac Everett, Chapman Coleman, Wm. E. Glover, James Speed, and Wm. H. Pope. I would ask, are these men to be tampered with—our judges, eminent lawyers, leading merchants, men of the first note in our community, as Dr. Caldwell justly remarks—some of them, *not*, as his phrase is, "*fierce*," but consistent, Christians? It would seem a waste of time to answer such a charge involving such a body of men; and the more so as in making it Dr. Caldwell has not thought it worth while to refer to a single fact in proof, has not adduced the shadow of an argument, gives not the name of a solitary witness, but seems to flatter himself that the statement will be believed on the naked authority of his name. I might content myself with giving to it an unqualified denial, and defying its author to show anything in its support; but I will do more than this—I will show its utter absurdity.

The new Board of Trustees took possession of the school in April, 1846, and among their "by-laws" they ordained that a professor was superannuated at the age of sixty-five. This was regarded by Dr. Caldwell, and by everybody else at the time, as a delicate way of expressing to him the opinion of the board that the period had arrived when he ought to retire. This opinion was plainly expressed by several influential members of the board to different members of the faculty, and the belief was very general that if he did not resign he would be removed. This is known to all Louisville, except Dr. Caldwell. The faculty took steps to save him from this mortification; for, although even then a majority of the members agreed with the board as to the policy of his resigning, they were pained at the thought of his being forcibly expelled, and accordingly requested Dr. Miller and myself to confer with certain trustees, and satisfy them, if we could, that he ought not at that time to be disturbed. That season no movement was made by the board against him.

In the winter of 1847, toward the close of the session, a member of the Board of Trustees, whose name is known to Dr. Caldwell, said to me that he believed it was the determination of the board to move against Dr. Caldwell in the spring if he persisted in holding on to his chair. He added, that the measure would be unpleasant, and that Dr. Caldwell ought to save the board the necessity of resorting to it by sending in his resignation at the close of the course. And he concluded by asking me to go to Dr. Caldwell and urge him to resign. Another member, about the same time, expressed the same opinion, and made a similar request. The professors held frequent consultations on the subject, and their belief was nearly unanimous that Dr. Caldwell owed it to himself and to the best interests of the school to resign.

It was under these circumstances, and explaining them all to him, that I called upon Dr. Caldwell and expressed a hope that he would gratify the wishes of his friends in the board and in the faculty. I added, that, if he were my father, my advice to him would be the same. In the course of our interview, he asked me who was thought of as his successor? I replied, Dr. Bartlett. He refused to listen to my advice; and shortly afterwards learned that he was accusing me in the streets of having intrigued with the board to remove him.

He visited the members of the board personally, represented to them that the receipts of his chair were necessary to the support of his family, but that in two or three years he would be comfortable, and would then resign. His appeal was successful; he was suffered to remain. One session passed, and a second was passing away. On the first night of January, 1849, his colleagues had a meeting. His chair was the subject of conversation, and the impression with them was unanimous that his longer continuance in it was out of the question. It was understood that the Board of Trustees entertained the same opinion. It was agreed, by all, that Dr. Bartlett should be recommended to the board as his successor, provided that gentleman would accept; and the Dean of the Faculty was directed to open a correspondence with him on the subject.

I have another fact to state. Some years since the faculty adopted a resolution to this effect: "That whenever in the judgment of a majority of the faculty the interests of the school were suffering by the connection of any professor with it, such professor, on being requested thereto by his colleagues, should resign without an appeal to the trustees." Dr. Caldwell voted for this resolution and advocated it warmly. With such a law in the faculty, I ask, what reason could any professor have to seek the interposition of the board? It was ample, specific, and fully met Dr. Caldwell's case. There was no appeal from it. He had aided in passing it, and was bound by every sacred obligation to obey it.

In 1847, near the close of the session, the faculty passed a resolution in respect to superannuation, by which Dr. Caldwell felt himself instructed to resign. He was assured that his colleagues entertained the opinion that he ought. In 1849, some weeks before he was removed, a letter was addressed to him, signed by all his colleagues, requesting him to send in his resignation to the board.

Thus, I have shown that the present Board of Trustees came into office convinced that Dr. Caldwell ought to retire from the school—that one of their first acts was to pass a law expressive of that conviction—that, even at that time, nearly all his colleagues, if not every one, believed the same thing—that, two years before he was removed, influential members of the board, friendly to him, requested me to go to him, as his friend, and *urge* him to resign—that by a resolution of the faculty he was instructed to resign three years ago—and that, so far from expecting to succeed him in his chair, Dr. Bartlett was the individual always mentioned by myself and by my colleagues as his successor. And now, in view of all these facts, I ask, what becomes of this last charge of my old friend [illegible]

I leave the decision to the candid reader, and here close my reply. So unprovoked, so unjust, so scurrilous an attack as Dr. Caldwell has made upon me would have justified, I know, in the opinion of many, something more than a vindication of myself. But I am not going to retaliate. I have been Dr. Caldwell's friend. I have written much in his defense. In all his late troubles it was my wish to protect him, and, with my colleagues, I endeavored to save him from the worst enemy his fame has in the world—we sought to save him from himself. Nor do I believe that he has a single judicious friend who does not, this day, regret the infatuation which led him to reject our friendly counsel.

---

After the foregoing reply to Dr. Caldwell was in type, a second attack upon me, far more scurrilous than the first, appeared in the Louisville Democrat under his name. In this latter effusion my old friend has cast off all restraints and broken through all the observances that regulate the intercourse of gentlemen. His blind, impotent rage knows no bounds. His ribaldry is like the scolding of a fishwoman. All the sanctities and decencies of life, all that men hold most sacred and dear, he violates and seems to contemn. One not acquainted with the style in which it has been his custom to denounce his enemies might doubt the soundness of his mind while reading this vulgar outpouring of his malice, so much is it like the railing of the insane man against his best friends. And why all this? the reader will naturally inquire. Because, as he declares, "I had the folly and impudence," in my valedictory lecture to my class a year ago, "to pretend to be one of the founders of the Louisville Medical Institute." I claimed to be one of the six professors who taught its first class, and to have worked hard in the places assigned me. Surely a most flagrant assumption! Most unparalleled impudence! A most ample apology for a foul stream of personal abuse running through nearly three columns of a newspaper!

Painful as it is to continue this controversy, I nevertheless feel constrained to take some notice of this article. I shall do so very briefly, and, notwithstanding its base character, shall endeavor to do so temperately. Fortunately the preceding reply furnishes a sufficient answer to most of its pitiful accusations, and I may now pass them by unnoticed.

The reader who has any knowledge of what has transpired in the politics of Western medical schools, in the last thirteen years, will feel some surprise at learning that Dr. Caldwell has brought himself to the point of asserting that I was the only professor "expelled" from the Transylvania Medical School in the disruption of 1837. He admits that this assertion is in the face of all that has heretofore been said and believed on the subject; nevertheless he ventures to make it. He is a bold man, or else he has a very short memory. Can he have forgotten that the Board of Trustees of Transylvania University kept a record in stated that *he* was "expelled" and he alone of all the professors—that he was expelled, however unjustly, at least irrevocably, and, I believe, unanimously? Can he have forgotten that after his expulsion the faculty was dissolved, and dissolved with the avowed object of effecting a reconciliation of differences among the remaining professors? Can he have forgotten his own letter to me on the subject, in which he says: "Cooke and yourself are to be reinstated only at the solicitation of Dudley, and I, from having sinned beyond forgiveness, am dismissed?" Has he forgotten the letter of Mr. Wm. A. Leavy, one of the board, and one of the best of men, written to me during the progress of those events and published at the time, stating that Mr. Wickliffe, chairman of the board, avowed, while offering the resolution dissolving the faculty, that "a leading object with him (Mr. W.) was the hope of seeing harmony restored among the professors, and particularly his wish from his view of Dr. Yandell's talents, character, and influence, that the college should retain him as a professor?" Has he forgotten what Mr. Leavy adds, namely, that "in these sentiments, which were also avowed by one or two other members, I have no doubt *the great body of the board sincerely participated?*" That I could have been reinstated, Dr. Caldwell has never entertained a doubt; that I was not reinstated he knows perfectly well was because Dr. Dudley was fully apprised of my determination not to accept again. What did Dr. Caldwell counsel at the time? In a letter to me dated Louisville, March 31st, 1837, he says: "The true method to mortify Dudley and his board (for they *are* his) is for Cooke, Short, and yourself to allow them to make an open offer of reinstatement, and reject their offer without comment. This will also crush the school beyond all hope of recovery; for if you all thus reject, no man of any standing will afterwards accept." But Dr. Dudley was a little too wary to be caught in any such trap. He was firm against the wishes of the board, against reconciliation and against a reinstatement, which was to be thus turned against the school.

As to all that Dr. Caldwell alleges concerning my "tremulous vacillation," my "doubt and indecision," in respect to accepting the chair tendered to me in the Medical Institute of Louisville, I have only to say that my letter of acceptance to the President of the board of managers, written without a moment's delay—that all my letters to Dr. C. about that period, and all his letters to me—stamp falsehood upon its face. Moreover I may be permitted to add, the allegation harmonizes very completely with my old friend's conception of me at the time as being the man "to ride on the whirlwind and breast the troubled wave!"

But there is one charge in Dr. Caldwell's second attack which I must notice more seriously. It is to this effect: that while I never brought a student to the school by my "lectures," or by "my height, dignity, or weight of character," I have brought "pupils to it by artifice and management;" that I have "detained here, by artifice, in their passage to the North and East, many pupils" whom I could "never have at- [illegible]

I confess I am at a loss with what epithets to brand this statement as coming from Dr. Caldwell. It is one of the worn out calumnies of two or three men whose names shall not pollute this page—of men who have oftener traduced Dr. Caldwell than myself, and whose abuse he has as often declared he took to be the highest eulogy they could bestow upon him. It is this miserable, threadbare, most contemptible slander which, with real or affected indignation, he has himself a hundred times scouted, that now, the bitterness of his hate and without any show of shame or remorse, he comes forward to vamp up and start afresh upon his authority. I will not trust myself to speak of such conduct in the terms it deserves. But suppose I had acted in the manner that these men and Dr. Caldwell say that I did, where does he place himself by becoming my accuser? Need I remind him of the maxim of the law in such cases? Was he not, at the time I was acting as he alleges, my colleague, and the most laudatory of colleagues? And if I inveigled students into stopping at our school, did not a fair share of the profits of the transaction inure to him? For every student thus "detained by artifice," did not *fifteen dollars* (a big sum in his eyes) find their way into his pockets? Out upon such a man! If his friends have any prudence, they will compel him henceforth and forever to abjure pen, ink, and paper. It is plain that he has forgotten everything—he has even forgotten that he belongs to an honorable profession.

Dr. Caldwell's account of the circumstances under which I was transferred by the Board of Trustees to the chair of physiology and pathological anatomy is of a piece with all his other statements; it is not necessary that I should characterize it. I have shown how easy it is for him to contradict himself. If he should live and be permitted to write a few years longer, he will, in all probability, give the lie to this as he has done to so many of his other fabrications. The facts of the case referred to are simply these: Dr. Bartlett having declined to accept the chair in question, it became necessary to consider what disposition should be made of it in the event of its being vacated. Various plans were proposed and discussed. Some thought that a chair, which had fallen into such disrepute by reason of the manner in which it had been long filled, might with great propriety be abolished. While the several schemes, and this among the rest, were under discussion, the board concluded to settle the matter in their own way, according to the best light before them, and so elected me to the chair of physiology and pathological anatomy, and elected Dr. Silliman professor of chemistry. There was no nomination in either nor did either of us ask for a nomination. Th was the board's, and I presume the board are fied wh the verdict pronounced by the public their proceedings. The school has gone on flou ing—the class is larger than the last—the most monious session known in the history of the in tion is just drawing to a close, and the decision c students, the best judges by far in the case, has abroad to the world. That decision, I shall be doned for saying, is all that trustees or profe could desire. But, if it had been other than it must say, I should not have given the boar trouble to "vacate" my chair, nor my colleague pain to remind me of "faculty rules;" but I shou not with cheerfulness, yet, I trust with dignity, retired to that "farm in Tennessee," of which old friend writes so much, into the bosom of society which has known me from my boyh and into the ranks of my profession, where I sh deem it neither a disgrace nor a hardship to pas days that may remain to me of active life.

My aged adversary warns me, in conclusion, the grave, which cannot be far removed from and may be near to us both, is not to silence his umnies. From that resting-place, to which most look as a place of peace, he threatens to pursue with his malice, and, in a work "designed to be thumous," to "suspend me on a gibbet," or in upon me the tortures of Marsyas. My poor old fri Posterity, I apprehend, will little heed his wo past or to come, his bickerings, his hatreds, or loves. But, if the threatened work should appe my day, I shall not despair of being able to dis as easily of his posthumous as I have found it to his living slanders; and if it should be delayed til after I am gone, I have no fears that I shall leave behind me those who will fully vindicate memory against the aspersions of a man who villified every associate he ever had, and now, at close of an extremely long life, among all his nu ous colleagues, living or dead, cannot count one gle friend.

And now I hope I am done with Dr. C well. He must indeed be very outrageous to d me out again. He has nothing to do but to n his spleen—to abuse his old friends before dinner an appetite and after dinner for digestion, but I h other and more profitable occupation than reply to the effusions of such an adversary. He must cuse me, therefore, and not take it as any mar disrespect, if I decline any further controversy w him.

